# Ramsay Hunt Syndrome: A Rare Complication of Herpes Zoster Infection With an Incidental Finding of Submandibular Hemangioma

**DOI:** 10.7759/cureus.66020

**Published:** 2024-08-02

**Authors:** Vedant M Dhatrak, Swapnil Mohod, Sourabh B Shinde, Vikrant V Jadhav

**Affiliations:** 1 Oral Medicine and Radiology, Sharad Pawar Dental College and Hospital, Datta Meghe Institute of Higher Education and Research, Wardha, IND; 2 Orthodontics and Dentofacial Orthopedics, Sharad Pawar Dental College and Hospital, Datta Meghe Institute of Higher Education and Research, Wardha, IND

**Keywords:** hemangioma, corticosteroids, facial nerve paralysis, herpes zoster virus, ramsay hunt syndrome (rhs)

## Abstract

Ramsay Hunt syndrome is a rare viral condition that develops from the varicella zoster virus that affects the face's geniculate ganglion. It has been defined by a herpes zoster oticus, which can be associated with further cranial nerve lesions and acute peripheral facial nerve palsies. In this case, we present a 73-year-old female patient who presented to the outpatient department (OPD) with unbearable pain in the lower left cheek that she had been experiencing for the last four days. The reported pain was continuous and could be described as deep-aching and burning. Facial swelling was observed in relation to the lower lip, especially in the vermillion area; there was ulceration, paralysis seen on the left face, and swelling on the submandibular region. Intravenous corticosteroids and antiviral drugs were administered to her for seven days as an association therapy. In this report, the authors want to stress the necessity of using adequate clinical examination and early intervention in the case of the Ramsay Hunt syndrome.

## Introduction

Ramsay Hunt syndrome (RHS) was named by neurologist James Ramsay Hunt in 1907. This is a rare ailment for which, in the beginning, the patient presents with symptoms of numbness and weakness in the facial muscles on one side of the face; the patient is then characterized by a varicella-zoster rash on the same side of the face. It is secondary to recurrent herpes zoster, which is due to the varicella zoster virus (VZV) but at the geniculate ganglion. Reactivation and replication of varicella-zoster virus cause herpetic inflammatory lesions to emerge from the ganglion to dermatomes related to the relevant ganglion. Therefore, the sign on which to focus in a patient with herpes zoster is pain and distribution of a definite rash primarily within the dermatomal zone [1,2]. Elderly people, people under stress, and people with compromised immune systems are commonly affected by herpes zoster (HZ) [3]. These anastomoses could include the facial, trapped in the stylomastoid foramen, the trigeminal, the glossopharyngeal, and the vagal or the cervical in the neck sinus, and thus herpetic lesions might worsen because of the interconnection of the cranial nerves. Diagnosis of herpes zoster oticus is often attributed to skin lesions; however, there are many neurological conditions, such as vertigo, tinnitus, hearing impairment, and vomiting, to name a few. [4,5]. In Hunt's zone, the pain could be severe, may be localized, or could radiate to the head, neck, face, or ears due to acute herpetic neuralgia. I understand that the sympathetic nervous system and this neuralgia are connected.

Thus, lacrimation, nasal congestion, and salivation can occasionally be brought on by pain [6]. The primary diagnostic criteria for Ramsay Hunt syndrome are the neurological examination, clinical symptoms, and history. Polymerase chain reaction tests of the sera collected from the patients revealed that both the ear fluid sample and eye swab samples were carrying DNAs of Varicella Zoster virus [7, 8]. Histopathological examination in (RHS) cases identified round cell infiltration within the facial nerve sections surrounding the streets through a perivascular, perineural, and intraneural manner. VZV can also impact the VIII cranial nerve, resulting in vestibulocochlear symptoms. To manage such rare cases, medical and surgical intervention is used [9]. When obtaining a higher multiplication rate, the cells that make up the lining of the oral cavity, namely endothelial cells, can develop benign oral tumors known as oral hemangiomas (OHs). Combined, roughly, hemangiomas of the head and neck region comprise between 60-70% of all the reported cases of hemangiomas, which are rare but more prevalent in the palate, lips, tongue, and buccal mucosa [10]. It has also been reported to involve the maxilla and, to a lesser extent, in the mandible; there are central hemangiomas, and in the muscles of mastication, mainly the masseter and other muscles of mastication, the so-called intramuscular hemangiomas.

## Case presentation

**Figure 1:**
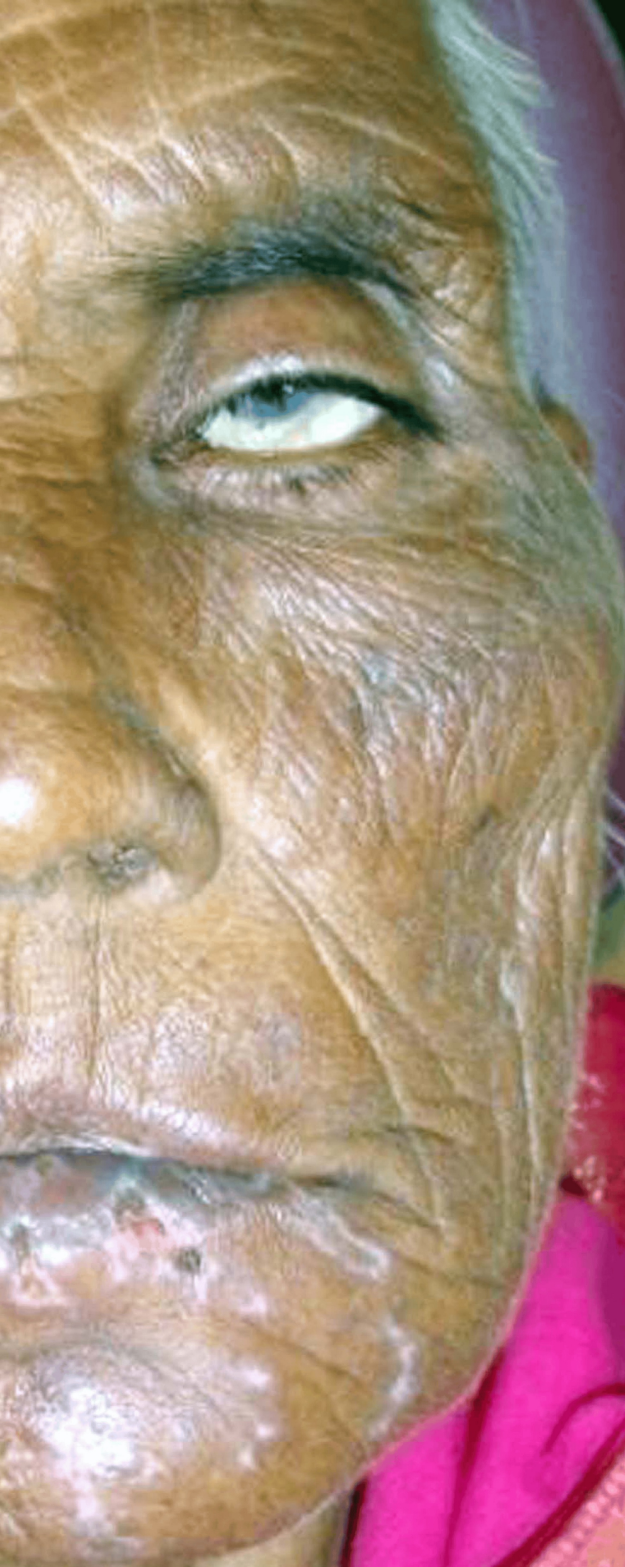
Multiple healing ulcers were seen on the left chin and parasymphyseal region, drooping of the left corner of the mouth was seen, and the patient was not able to close the left eye, which shows a positive Bell's sign

A 73-year-old woman arrived at the outpatient department (OPD) complaining of excruciating discomfort on her lower left cheek, which had been there for the last four days. The pain was constant, deep-aching, and burning in nature. On extraoral examination, the face is asymmetrical due to diffuse swelling on the left side of the lower jaw. TMJ is bilaterally smooth and synchronous. Lymphadenopathy was evident in both the right and left submandibular region as a single submandibular lymph node was palpable in both the left and right submandibular region of size 0.5 cm x 0.5 cm and non-tender. Ulceration is seen on the vermillion border of the lower lip on the left side. Multiple healing ulcers were seen on the left chin and parasymphyseal region. Drooping of the left corner of the mouth was seen, and the patient was not able to close the left eye (Figure 1).

**Figure 2:**
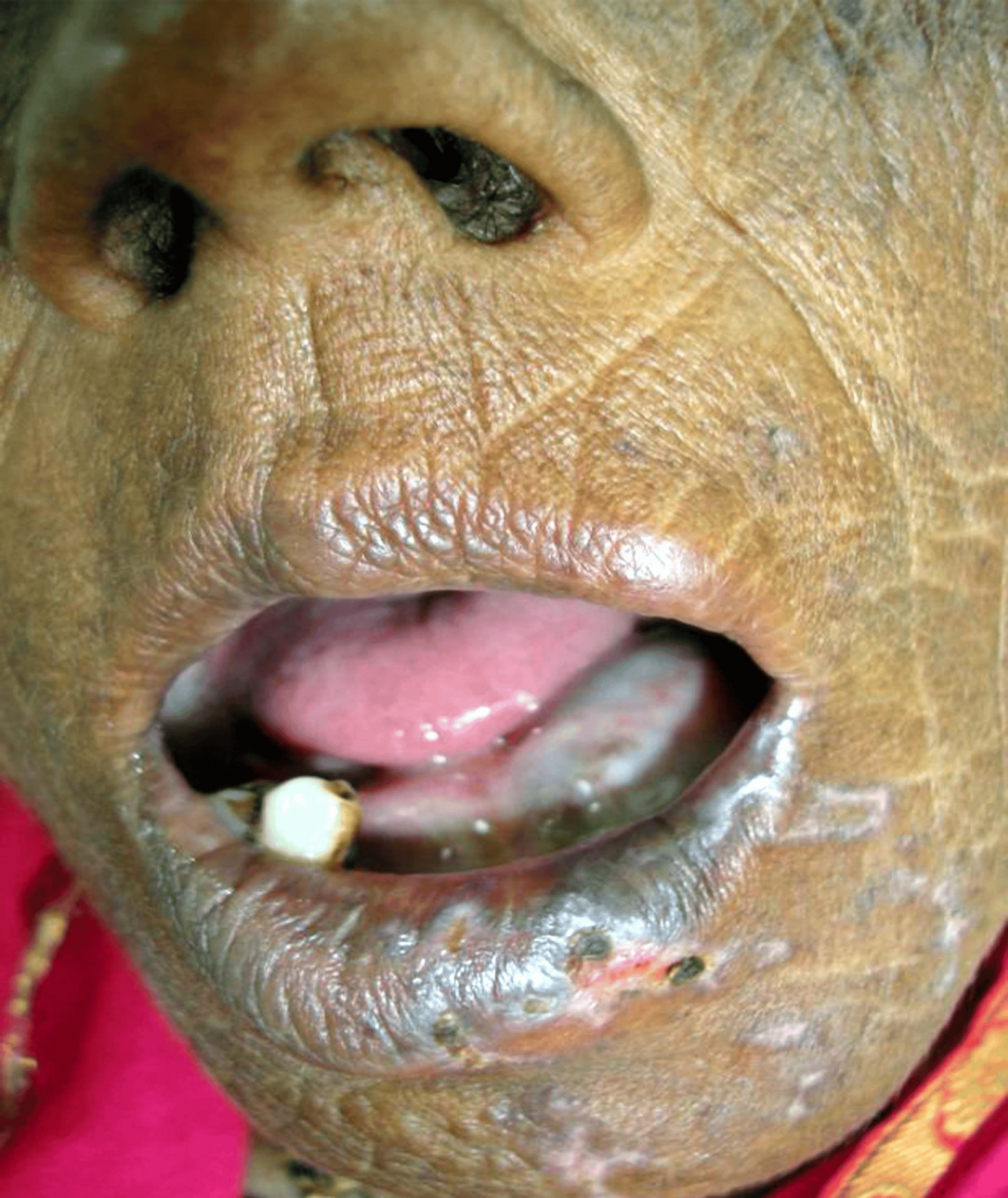
On intraoral examination, a bluish swelling was present on the lower left side of the floor of the mouth, which was soft in consistency and non-tender in nature with no bleeding or pus discharge present on manipulation

On intraoral examination, a bluish swelling was present on the lower left side of the floor of the mouth, which was soft in consistency and non-tender in nature, with no bleeding or pus discharge present on manipulation. The lower alveolar ridge was resorbed severely as the teeth showed mobility. Upper right second molar grade 2 mobile, with the presence of severe gingival recession that was generalized. Moderate stains and calculus were seen with generalized gingival inflammation (Figure 2).

**Figure 3:**
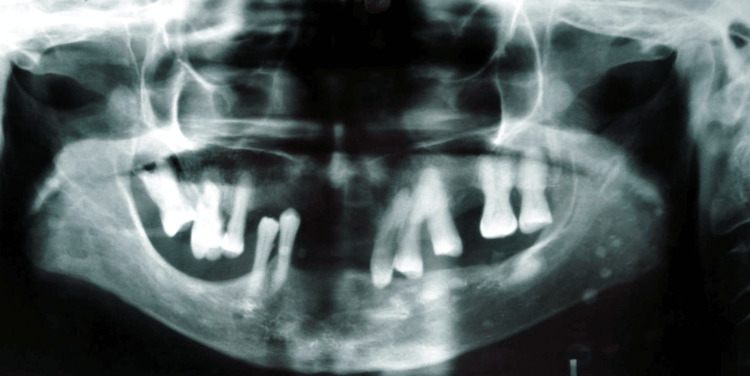
Panoramic view showing multiple Bull's eye-like radiopacities in the lower left back region of the jaw suggestive of phleboliths

On radiological examination, a panoramic showed multiple Bull's eye-like radiopacities in the lower left back region of the jaw suggestive of phleboliths (Figure 3).

A provisional diagnosis of HZ infection involving the trigeminal nerve with sublingual hemangioma was made after the patient's complete clinical and radiological history was taken. Treatment of this rare lesion lies in the following: Treatment is supportive and aims to relieve the patient's symptoms and pain. First of all, medication therapy is administered like antiviral medication comprising of acyclovir (Zovirax) to help decrease the viral infection of herpes zoster. Analgesics (Ibuprofen) and anticonvulsants (gabapentin) were simultaneously given to relieve the facial pain and nourish the nerve.

To stabilize the mental state and for a proper slip cycle, tricyclic antidepressants (amitriptyline) were given. Steroids (prednisone) were given to reduce inflammation and facial swelling of the area involved. We were also treating facial nerve paralysis concurrently, such as facial massage and exercises, physical therapy, and electrical stimulation of the nerve, to calm the nerve and face muscles. Facial paralysis also affects the eyes; the serious drooping of the eye signs was observed, so eye treatment was provided by the health practitioner to the patient. Eye drops for lubricating the eye site and a patch to protect the eye cornea were provided. Other treatments can also be given that are used as possible antiviral eye drops and (VRT) vestibular rehabilitation physical therapy (for balance disorders, vertigo). Treatment and support for the patient ought to include sufficient amounts of rest, adequate fluids and water metabolism, as well as a healthy diet that sustains the body weight. The patient was followed up after three weeks.

Post-treatment Ramsay Hunt syndrome also involves the treatment of residual symptoms, preventing the reoccurrence of the disease or the occurrence of other related diseases. Patients should follow the follow-up appointments regularly and visit their doctor to monitor progress and address concerns. They should not stop medication therapy such as antiviral, anticonvulsant, and steroids until the doctor directs for proper outcome. Patients should perform facial exercises regularly to improve facial strength and symmetry lubrication of the eye, and regularly use an eye patch to protect the cornea and eye site. Some lifestyle modifications should be explained to the patient after treatment, such as getting plenty of rest, maintaining a healthy diet, and reducing stress for the proper outcome of the treatment. Most importantly, counseling should be offered to patients on follow-up, such as addressing emotional and psychological aspects of RHS, like depression or anxiety. Patients should be monitored for complications and watched for signs of corneal ulcers, facial weakness, or other complications. Thus, by understanding and practicing these post-treatment maintenance tips, you would be able to prevent future reoccurrences of Ramsay Hunt syndrome and its complications.

## Discussion

RHS is a rare disease, and based on scholarly research, it has been estimated to occur in 0.3 - 18% of acute atraumatic facial palsies. The best known of these is Bell's palsy, which is the most frequently reported cause of RHS [11]. These are ipsilateral peripheric facial paralysis, acute ear pain, known as herpes zoster oticus, and vesicular lesions at the external ear. It arises from the reactivation of the varicella zoster virus (VZV), which is latency in the geniculate ganglion. Varicella zoster virus is categorized as a DNA virus and is associated with sporadic and recurrent disorders.

It affects individuals across all age groups; however, it primarily affects the elderly. Prodromal symptoms include neuropathic pain, headaches, malaise, and impaired sleep [12]. The symptoms of RHS include cranial nerve V peripheral facial palsy, acute pain of the ear (herpes zoster oticus), and vesicles on the auricle. It originates from the varicella zoster virus returning from a dormant state, having initially infected the geniculate ganglion. As described by Grose et al., RHS neuropathogenesis involves the fact that the virus can penetrate ganglion during diseases, such as chickenpox, due to the cells of sensory branches of the facial nerve, which is present in the mouth and ears. Therefore, when VZV is reactivated, it moves back down the FSN's sensory divisions, and the nearby divisions of the facial nerve become swollen due to this action, though not directly infected, leading to the condition known as facial palsy [13]. Histological examinations demonstrated perivascular, perineural, and intraneural aggregation of round cells in the facial nerve. VZV can also influence the VIII cranial nerve, which might result in vestibulocochlear symptoms [9].

The neurological examination, histological examination, clinical symptoms, and patient history are the main diagnostic criteria for RHS. Using polymerase chain reaction techniques, herpes zoster virus DNA can be identified in exudates such as ear scrapings, tears, saliva, blood mononuclear cells, and cerebrospinal fluid [7, 8].

At the moment, the principles of treatment are based upon the combination of corticosteroids and antiviral drugs, which have proven to affect the treatment of this disease. The most common dose of antiviral drug acyclovir 800mg four to five times a day is prescribed to patients. Murakami et al. compared the acyclovir 15 mg/kg per day parenteral dose in 32 patients and 800 mg four times a day in 48 patients, and they observed no significant difference between the two groups, so they concluded that the oral route of acyclovir is more practical because it required less time than the parental route [7]. When Ryu's study compared 202 patients with RHS and 155 patients with Bell's palsy, RHS showed more severe paralysis and fewer results of complete recovery; both the groups were treated with the same combination dose of antiviral and steroids, and he finally concluded that RHS showed poor results and more severity of facial paralysis and more facial weakness as compared to Bell's palsy. Therefore, they investigated the early diagnosis and treatment required to reduce the severity of this rare disease [14,15].

Since the incident finding of oral hemangiomas in this case has also been discussed, most oral hemangiomas are benign in origin and rapidly undergo total involution over time; therapy is not usually necessary treatment is advised, therefore, for OHs who exhibit difficulties speaking, eating, or breathing, as well as for the 10% to 20% of lesions that continue throughout puberty or adulthood. There are two types of current treatment options: surgical and medical (or interventional) [16].

## Conclusions

Ramsay Hunt syndrome is rare and, therefore, often has various signs and may manifest in different forms, as demonstrated in the present case. The risk of developing one or more of the complications of HZ requires careful attention to patients who present with symptoms of the condition, placing clinicians on high alert. It has been shown in previous studies that the outcomes of these patients will significantly improve if they are administered antivirals and corticosteroids at the beginning. The treatment of Ramsay Hunt syndrome depends on the degree of damage caused to the nerves, and it is crucial to diagnose an individual as soon as possible so that the treatment can begin immediately.
